# Profiles of physician motivation towards using virtual care: differences in workplace need fulfillment

**DOI:** 10.1186/s12913-023-10057-x

**Published:** 2023-10-16

**Authors:** Oksana Babenko, Adam Neufeld

**Affiliations:** 1 Department of Family Medicine, Faculty of Medicine & Dentistry, University of Alberta, Edmonton, AB Canada; 2 Department of Family Medicine, Cumming School of Medicine, University of Calgary, Calgary, AB Canada

**Keywords:** Virtual care, Healthcare workforce, Motivation, Wellbeing, Self-determination theory

## Abstract

**Background:**

Physicians appear to vary in their motivation towards using virtual care, but to what extent is unclear. To better understand this variance, which is important for supporting physician wellbeing and therefore patient care, the authors used self-determination theory’s (SDT) framework. According to SDT, different types of motivation exist, ranging from controlled to autonomous, that lend to differences in engagement, performance, and wellbeing. The authors aimed to determine: (a) if there were distinct groups of physicians based on their quality of motivation towards using virtual care, and if so, (b) how these groups varied in fulfillment of basic psychological needs (autonomy, competence, and relatedness) in the workplace.

**Methods:**

In March-August 2022, the authors collected quantitative, survey-based data from a cross-section of 156 family physicians in Alberta, Canada. The survey contained existing scales that measure types of motivation (autonomous vs. controlled) and basic psychological need satisfaction/frustration at work. Cluster analysis was used to explore profiles of physician motivation towards using virtual care, and analysis of variance was used to determine how each profile differed with respect to workplace need fulfillment.

**Results:**

With motivation towards using virtual care, three higher-order profiles of physician motivation were identified: *autonomous* (19% family physicians), *controlled* (16% of family physicians), and *ambivalent* (66% of family physicians). The three profiles differed significantly in terms of psychological need fulfillment at work.

**Conclusions:**

This study identifies specific profiles that family physicians currently fall into when it comes to motivation towards using virtual care. In line with SDT, findings suggest that basic psychological needs are fundamental nutrients for physicians to internalize and endorse the value of using virtual care in their practices. Implications for physician wellbeing are discussed.

**Supplementary Information:**

The online version contains supplementary material available at 10.1186/s12913-023-10057-x.

## Background

Physicians are using virtual care – telephone and video calls with patients – more and more frequently [1–3]. In fact, the recent Canadian Medical Association’s survey of more than 2,000 physicians (family physicians, specialists, and residents) revealed that 94% were currently using virtual care in their practices and 64% planned to continue or increase their use after the COVID-19 pandemic [1]. Yet, while there has been ample research on patients’ preferences and outcomes of virtual care use [4–7], physicians’ motivation towards using virtual care has received less attention.

According to self-determination theory (SDT) – a well-established theory of motivation – there are different types of motivation that exist along a continuum of autonomy, ranging from fully externally controlled to fully internally regulated [8, 9]. People therefore tend to engage in activities out of importance or interest (autonomous motivation) or because of external and/or internal pressures (controlled motivation) [8, 9]. The quality of motivation is important because it will directly shape people’s engagement and performance in the activity, and also their perceived stress and psychological wellbeing [8–10]. People may also have combinations of autonomous and controlled motivations towards certain tasks or activities, however, resulting in groups of individuals with nuanced differences in their functioning and mental health [10].

SDT-based research indicates that people’s quality of motivation and wellbeing is facilitated or hindered to the extent that the environment (e.g., workplace) supports their basic psychological needs [9, 11, 12]. These needs are autonomy (the need to feel that one’s actions are self-chosen), competence (the need to feel effective and capable of mastery), and relatedness (the need to feel connected to significant others) [9]. With the onset of the COVID-19 pandemic, physicians had no choice but adopt virtual care and rapidly change the way they practiced medicine (lack of autonomy). Furthermore, virtual care lacks physical observation, which supports diagnostic clarity [13], and this can add to ambiguity in managing patients, making physicians feel less effective (lack of competence). Many physicians also feel that virtual care hinders the therapeutic alliance with patients due to its lack of human contact (lack of relatedness) [13–15]. Unsurprisingly, studies have shown that physicians find virtual vs. standard care less professionally satisfying [16–19].

While the virtual healthcare and SDT literature is scarce, several articles do support the above concerns. For instance, Keenan et al. [4] found that virtual care supported patients’ basic psychological needs, but that physicians perceived more opportunities for virtual care to hinder than support those needs. Various themes have also been identified around virtual care and healthcare providers’ basic psychological needs, including pros and cons of scaling, impact of technology on autonomy and clinical competence, and quality of patient-provider relationship [20]. Another study compared the healthcare climate in traditional vs. virtual visits with family doctors. Even after controlling for patients’ preferred type of visit, virtual encounters came out as less supportive of basic psychological needs within the patient-doctor relationship [21]. Given that patient preference was not driving the results, the authors attributed their results to physician-related factors involved in self-determination [21].

The authors of these studies all emphasized the need to consider medical professionals’ perceptions about virtual care, to help optimize its integration into clinical practice. However, what has not been studied is physicians’ quality of motivation towards using virtual care, and in particular, whether there are distinct profiles of motivation (e.g., autonomous vs. controlled vs. mixed), and how physicians in each profile might differ psychologically. Investigating this is important because it can shed light on how virtual care can support or hinder physician wellbeing and job satisfaction, and what physicians need to maintain the delivery of high-quality patient care. In this study, we focused on family physicians, and aimed to determine: (a) if there were indeed distinct groups of family physicians based on types of motivation towards using virtual care, and if so, (b) how these physician groups differed in their basic psychological need fulfillment at work.

## Methods

### Participants and procedure

A cross-section of Alberta family physicians was invited to complete an anonymous online survey, containing demographic questions and two previously validated scales (see Measures and Appendix 1). Invitations were disseminated via list serves, academic newsletters, Alberta Medical Association primary care networks, and the Alberta College of Family Physicians and Well Doc Alberta websites. Invitations to participate contained information about the study, an ethics-approved consent form, and a link to the survey. Because the survey was posted on the above list serves and websites, we were unable to calculate a response “rate” per se; in the Results section we report the total number of physicians who completed the survey. Participation was voluntary, and informed consent was implied if they completed the survey, which also contained the study information letter. All were assured their confidentiality would be maintained. This research was approved by the Human Research Ethics Boards at the University of Calgary and University of Alberta. The data were collected from March to August in 2022.

### Measures

*Motivation towards using virtual care.* We used the 24-item Comprehensive Relative Autonomy Index (C-RAI), which measures the type of a person’s motivation towards engaging in some activity, along SDT’s autonomy continuum [22] It has been validated in samples of university and medical students, with high reliability values [22–25]. The C-RAI comprises six motivation subscales – three “controlled” and three “autonomous”. From least to most self-determined, these are: amotivated (acting without reason or volition), external (acting based on punishments or incentives), negative introjected (acting to avoid negative feelings, such as guilt or shame), positive introjected (acting to experience positive feelings, such as pride), identified (acting out of perceived importance and value), and intrinsic (acting based on interest or joy) [22, 23]. In this study, the C-RAI was used to measure how controllingly vs. autonomously motivated physicians were towards using virtual care in their practices. Participants were asked to indicate how true various items were for them, on a scale from 1 (not true at all) to 7 (very true). Higher scores indicate a stronger motivation of that type.

*Workplace need satisfaction and need frustration*. We used the 24-item Basic Psychological Need Satisfaction and Frustration Scale (Work Domain) [26], which measures a person’s level of satisfaction and frustration of their needs for autonomy, competence, and relatedness in the workplace. It has been validated and widely used among different populations with high reliability values, including academics, employees, and supervisors [26, 27]. Participants were asked to indicate their level of agreement with various items, corresponding to each need, using a scale from 1 (strongly disagree) to 7 (strongly agree), based on the last 4 weeks. There are six subscales in total – three for need satisfaction and three for need frustration – and higher scores indicate greater satisfaction or frustration of that respective need.

### Analyses

Analyses were performed in SPSS 26.0 (SPSS Inc, Chicago, IL). Mean imputation was used whenever at least 50% of scales were completed. We computed descriptive statistics and Cronbach’s alpha coefficients for each measure, which were all satisfactory. All continuous variables were checked for distribution normality based on skewness and kurtosis values, as well as linearity of relationships using scatterplots. We then performed two *k*-means cluster analyses to identify the smallest possible number of distinct clusters within the sample, based on the lower-order motivations (amotivated, external, negative introjected, positive introjected, identified, and intrinsic), and the higher-order motivations (controlled and autonomous). Prior research has shown that sufficient statistical power can be achieved with relatively small samples (*n* = 20 per subgroup), provided cluster separation (i.e., effect size) is on the larger side [28]. We therefore used these criteria for the cluster analysis in this study.

To conduct the cluster analysis, we first standardized all study variables, then determined the optimal number of clusters by exploring the clustering procedure for a range of 0 to 10 clusters. We assessed the validity of the cluster solution by assessing cluster tendency, the cluster iteration history, and the analysis of variance (ANOVA) statistics. Stability of the model was assessed by performing a double-split cross-validation procedure [10]. We used chi-square to test for cluster differences in physician gender, age, educational background, ethnicity, employment status, years in practice, and daily use of virtual care. We then used a one-way ANOVA to determine whether the resulting clusters of physicians differed in their satisfaction/frustration of autonomy, competence, and relatedness at work. Either a Bonferroni or Games-Howell correction was used in post-hoc pairwise comparisons when variances were equal or unequal, respectively. For all comparisons, we computed the associated Cohen’s *d* effect sizes, where 0.2 is small, 0.5 is medium, and 0.8 is large.

## Results

### Sample characteristics and variable relationships

One hundred and fifty-six family physicians participated in this study. The sample was diverse in demographic characteristics and the use of virtual care in practice (see Table 1). We examined bivariate associations between the demographic, motivation, and need-related variables. Higher frequency of virtual care use was associated with lower relatedness satisfaction at work (*r* = − .29, *p* < .01). Point biserial correlations indicated that male family physicians used virtual care more often (*r* = .33, *p* < .01) and had lower relatedness satisfaction at work (*r* = − .26, *p* = .01), compared to female family physicians. Results also showed that years in practice was positively associated with autonomous motivation towards using virtual care (*r* = .22, *p* = .03). Physician age, employment status, education, and culture/ethnicity had no significant relationship with the motivation and needs variables. Table 2 shows the correlations between the motivation and needs variables, which were all in the expected directions based on SDT.

**Table 1:** Sample characteristics (*N* = 156 physicians)

		n (%)
**Gender**	Female	110 (71)
	Male	41 (26)
	Non-binary/Other	3 (2)
	Prefer not to answer	2 (1)
**Age**	30 or under	2 (1)
	31–40	59 (38)
	41–50	35 (22)
	51–60	37 (24)
	61 or over	23 (15)
**Ethnicity**	Caucasian	100 (65)
	Latino/Hispanic	4 (3)
	Asian	38 (24)
	Indigenous	2 (1)
	Two or more	5 (3)
	Other/unknown	5 (3)
	Prefer not to answer	2 (1)
**Education**	Natural sciences	108 (70)
	Social sciences	13 (8)
	Languages	2 (1)
	Mathematics	2 (1)
	Business	6 (4)
	Education	10 (6)
	Caring profession	15 (10)
**Employment**	Full-time	114 (73)
	Part-time	34 (21)
	Seeking opportunities	4 (3)
	Prefer not to answer	4 (3)
**Years in practice**	5 or less	27 (16)
	6–10	32 (20)
	11–15	24 (15)
	16–20	19 (12)
	21 or more	54 (34)
**Use of virtual care**	Very infrequently	9 (5)
	Somewhat infrequently	42 (27)
	Occasionally	52 (33)
	Somewhat frequently	29 (19)
	Frequently	15 (10)
	Very frequently	9 (6)

**Table 2:** Means, standard deviations, and correlations for motivation and needs variables

	1	2	3	4	5	6	7	8
1. ASAT	(0.82)							
2. CSAT	0.59**	(0.91)						
3. RSAT	0.54**	0.64**	(0.87)					
4. AFRU	− 0.21**	0.04	0.05	(0.77)				
5. CFRU	− 0.17*	− 0.39**	− 0.20*	0.52**	(0.81)			
6. RFRU	− 0.11	− 0.16*	− 0.43**	0.35**	0.40**	(0.72)		
7. AM	0.18*	− 0.06	0.02	− 0.16*	0.11	0.04	(0.92)	
8. CM	− 0.33**	− 0.15	− 0.15	0.27**	0.10	0.25**	− 0.26**	(0.86)
*Mean*	4.31	5.19	4.93	3.56	2.06	1.79	2.50	1.87
*Std. dev.*	0.85	0.76	0.86	0.93	0.79	0.67	1.04	0.74

ASAT, autonomy satisfaction; CSAT, competence satisfaction; RSAT, relatedness satisfaction; AFRU, autonomy frustration; CFRU, competence frustration; RFRU, relatedness frustration; AM, autonomous motivation towards using virtual care; CM, controlled motivation towards using virtual care

* *p* < .05 and ** *p* < .01 Internal consistency reliability coefficients (Cronbach’s alpha) are shown in parentheses along the main diagonal

### Cluster analysis

Next, we explored whether distinct profiles of physician motivation towards using virtual care existed, based on SDT’s lower-order motivations (see Measures). Although several cluster solutions were statistically significant, few truly distinct profiles emerged and there was marginal, if any, difference among them in need fulfillment at work. We therefore explored profiles based on SDT’s higher-order (controlled and autonomous) motivations. Results indicated that a three-cluster solution best represented the data, with complete convergence. The ANOVA showed statistically significant differences between the three clusters, based on the grouping of controlled (*F* (2, 153) = 135.85, *p* < .001) and autonomous (*F* (2, 153) = 116.47, *p* < .001) motivations. Results of the double-split cross-validation assessment yielded very similar cluster solutions, thereby supporting the validity and stability of the results.

**Fig. 1:**
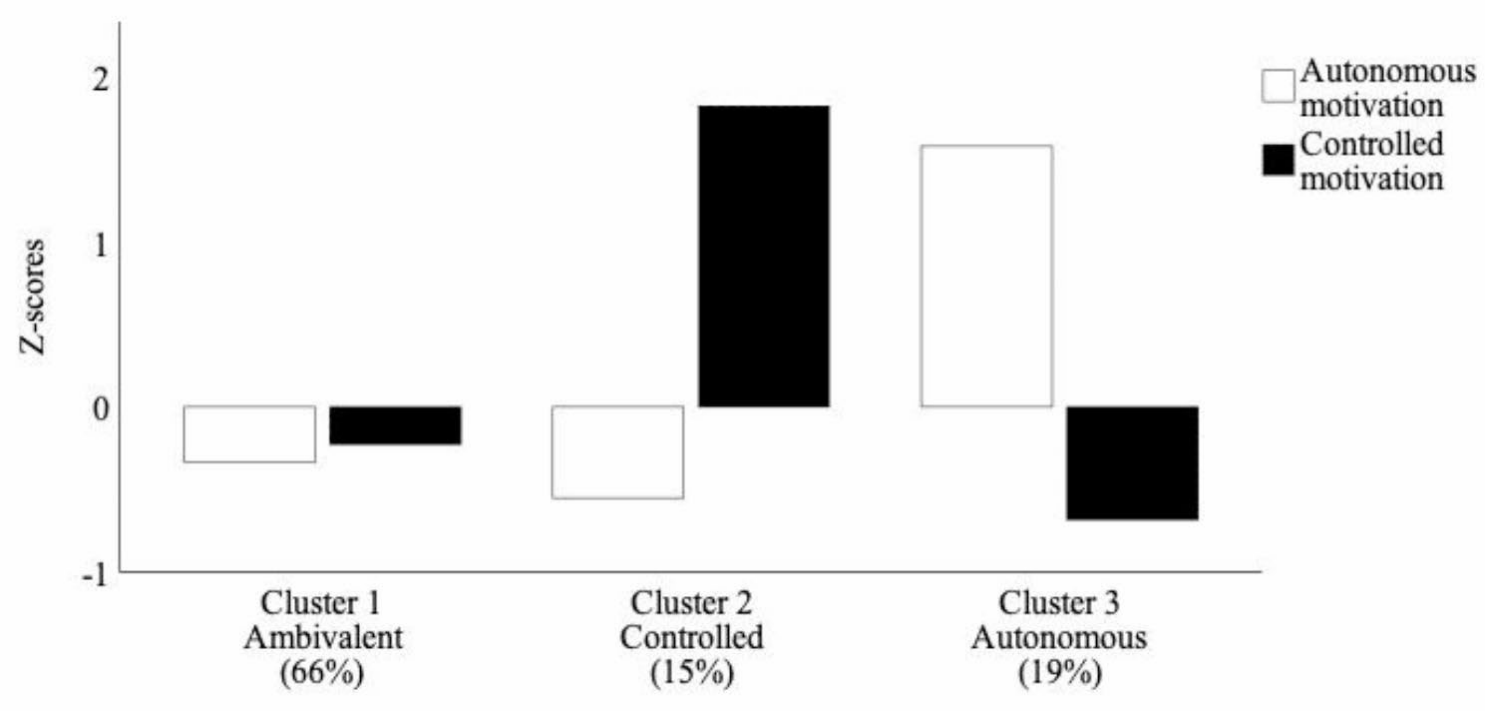
Physician profiles of motivation towards using virtual care

As seen in Fig. 1, we conceptualized the resultant three cluster profiles as: *ambivalent* – low in both controlled and autonomous motivation (*n* = 103 or 66% of family physicians), *controlled* – high in controlled motivation and low in autonomous motivation (*n* = 23 or 15% of family physicians), and *autonomous* – high in autonomous motivation and low in controlled motivation (*n* = 30 or 19% of family physicians). The results of chi-square tests confirmed that there were no significant differences between the three clusters in terms of gender, age, educational background, ethnicity, employment status, years in practice, and frequency of virtual care use (all *p*’s > 0.05). We therefore did not control for these variables in the subsequent analysis of between-cluster differences in workplace need satisfaction and need frustration.

### Between-cluster differences

As shown in Table 3, the ANOVA indicated statistically significant differences between the three clusters in their mean level of workplace autonomy, competence, and relatedness satisfaction, as well as workplace autonomy frustration. Post-hoc pairwise comparisons were thus performed to determine where the specific differences lay.

**Table 3:** Means, standard deviations, and ANOVA results by physician group

	Ambivalent motivation	Controlled motivation	Autonomous motivation			
	*n* = 103 (66%) *M (SD)*	*n* = 23 (15%) *M (SD)*	*n* = 30 (19%) *M (SD)*	*F*	*df*	*p*
*Need satisfaction*						
ASAT	**4.35 (0.69)** ^**a**^	**3.88 (1.02)** ^**b**^	**4.51 (1.07)** ^**a**^	4.06	2, 153	0.02
CSAT	**5.30 (0.58)** ^**a**^	**4.85 (1.11)** ^**b**^	5.05 (0.90)	4.03	2, 153	0.02
RSAT	**5.03 (0.72)** ^**a**^	**4.53 (1.27)** ^**b**^	4.89 (0.88)	3.16	2, 153	0.04
*Need frustration*						
AFRU	3.53 (0.94)	**3.96 (0.95)** ^**b**^	**3.33 (0.82)** ^**a**^	3.18	2, 153	0.04
CFRU	1.97 (0.67)	2.23 (1.11)	2.24 (0.87)	2.04	2, 153	0.13
RFRU	1.71 (0.52)	2.04 (1.09)	1.88 (0.68)	2.68	2, 153	0.07

ASAT, autonomy satisfaction; CSAT, competence satisfaction; RSAT, relatedness satisfaction; AFRU, autonomy frustration; CFRU, competence frustration; RFRU, relatedness frustration; M, mean, SD, standard deviation; df, degrees of freedom

Within each row, the means with different superscripts are significantly different from each other, i.e. a mean with superscript “a” is significantly different from a mean with subscript “b”

The *controlled* group reported the lowest autonomy satisfaction and the highest autonomy frustration at work. In terms of autonomy satisfaction, they differed significantly from the *autonomous* group (*MD* = 0.63, *SE* = 0.23, *p* = .02, *d* = 0.6) and *ambivalent* group (*MD* = 0.47, *SE* = 0.20, *p* = .04, *d* = 0.5). In terms of autonomy frustration, they differed significantly from the *autonomous* group (*MD* = 0.63, *SE* = 0.23, *p* = .03, *d* = 0.7). The *controlled* group also reported significantly lower competence (*MD* = 0.45, *SE* = 0.15, *p* = .02, *d* = 0.5) and relatedness (*MD* = 0.50, *SE* = 0.18, *p* = .04, *d* = 0.4) satisfaction at work, compared to the *ambivalent* group. There were no significant differences in competence or relatedness frustration between the three groups. Overall, the effect sizes of workplace need satisfaction, and autonomy frustration, were medium to large.

## Discussion

Guided by SDT, this study aimed to determine whether distinct groups of family physicians existed based on quality of motivation towards using virtual care, and how these groups might differ in terms of workplace need fulfillment. In this section, we discuss the correlational and profile analysis findings, their implications, study limitations, and future directions.

First, results showed that there was a negative association between frequency of virtual care use and relatedness satisfaction at work, providing support for existing literature [13–15]. Compared to female family physicians, male family physicians reported using virtual care more frequently, as well as experiencing lower relatedness satisfaction at work. Surprisingly, years in practice was positively associated with autonomous motivation towards using virtual care. Together, these findings suggest that (a) using virtual care more often may hinder family physicians’ need for relatedness at work, (b) how often family physicians use virtual care may vary based on their gender; and (c) family physicians who are further along in their career (but not necessarily older) may sense more autonomy in their decisions around using virtual care.

Using a *k*-means cluster analysis, we identified three motivational profiles of family physicians towards using virtual care. Most physicians fell into the *ambivalent* group, which was characterized by equally low levels of autonomous and controlled motivation towards using virtual care. In comparison, the other two physician groups had a more dominant type of motivation towards using virtual care – either controlled or autonomous. These three physician groups were all similar in terms of distribution by gender, age, educational background, ethnicity, employment status, years in practice, and frequency of virtual care use in practice. However, they differed significantly in their levels of workplace need fulfillment.

In line with SDT, physicians in the *autonomous* group reported the highest autonomy satisfaction and the lowest autonomy frustration at work. According to SDT, this is because individuals who endorse autonomous motivation towards an activity – in this case, towards using virtual care – will have internalized its value to a greater degree than individuals with competing motivations (*ambivalent* group), and those who perceive an activity as an imposition or threat (*controlled* group). Not surprisingly, the *controlled* group reported the lowest need satisfaction and the highest need frustration at work. Again, this presumably reflects a combination of amotivation (i.e., avolition) towards using virtual care and the various pressures family physicians face when using it – both external (e.g., meeting financial demands) and internal (e.g., not letting their patients down).

The *ambivalent* group of physicians (with low autonomous and controlled motivation towards using virtual care) may be “ambivalent” because they use virtual care potentially for time saving and due to patient preference. Hence, these physicians may dislike and/or feel pressured to use virtual care (i.e., external motivation) but also see it as useful and important (i.e., identified motivation). Why physicians in this profile reported significantly higher workplace relatedness and competence satisfaction is unclear. However, with frequency of virtual care use being equal, it might relate to how the *ambivalent* physicians use virtual care. For example, if these physicians feel uncomfortable managing certain ailments virtually, they may be quicker to convert those visits to in-person ones, which can be easier and more rewarding to manage. In other words, some skepticism about using virtual care, combined with selectivity about when to leverage it, may support higher competence and relatedness satisfaction for these physicians, compared to physicians whose motivation is of a dominant type (i.e., either controlled or autonomous).

The study has several limitations which can help guide future research. First, the sample is diverse in terms of demographic characteristics and the use of virtual care in practice; however, results are based on data from only family physicians in one Canadian province. Second, while the sample size and effect sizes were sufficient for the cluster analysis and post hoc comparisons [28], it is unclear whether the lower-order profiles (based on SDT’s six types of motivation) are truly absent or may have not been detected due to the relatively small subgroups. Larger scale studies are therefore recommended to confirm and extend our findings, both in family medicine and other medical specialties. Third, the data are based on self-report measures collected via surveys, which creates the potential for response bias. Additionally, some of the demographic questions (e.g., frequency of virtual care use) were created by the authors, and the duration of time that physicians used virtual care, based on the frequency that they indicated, was not accounted for. Accounting for both frequency and duration is therefore recommended in future studies.

The strengths of this study include the use of well-established measurement instruments that derive from sound theoretical evidence and collecting data from a broad group of family physicians from across Alberta. We also accounted for a range of demographic factors that were hypothesized to potentially confound results, with respect to physician motivation towards using virtual care. While the findings can help inform the literature base, conducting qualitative studies, including semi-structured interviews with practicing physicians, is warranted to further unpack physicians’ perceptions about virtual care, as well as its influence on their job satisfaction, wellbeing, and quality of patient care.

## Conclusions

This study provides initial insights into physician motivation towards using virtual care, along with some of its psychological consequences. The *ambivalent* profile appears to be the dominant type among family physicians when it comes to motivation towards using virtual care. Compared to the *controlled* group, and to a lesser extent, the *autonomous* group, this group of physicians reported significantly higher fulfillment of basic psychological needs at work. Together, results shed light on how virtual care can both benefit and hinder physician motivation, and how important the right balance is for optimal functioning. These findings help inform educational and training efforts in medicine, and help physicians understand their own motivation towards using virtual care and its impact on their professional wellbeing.

### Electronic supplementary material

Below is the link to the electronic supplementary material.

Supplementary Material 1

## Data Availability

The data analysed during the current study are available from the corresponding author on reasonable request.

## List of Abbreviations

SDT: Self–determination theory
