# Supplementary material for: Profiles of physician motivation towards using virtual care: differences in workplace need fulfillment
Source: BMC Health Serv Res. 2023 Oct 16;23:1101. doi: 10.1186/s12913-023-10057-x (PMC10580539; doi:10.1186/s12913-023-10057-x)
Supplement: Supplementary file 1 — Supplementary Material 1 [file 12913_2023_10057_MOESM1_ESM.docx]

**APPENDIX 1 – Demographic items and scales**

**Demographic items**

1. “What is your age?” (30 years or under; 31-40 years; 41-50 years; 51-60 years; 61 years or over; Prefer not to answer)

2. “How do you identify?” (Male; Female; Non-binary/Other; Prefer not to answer)

3. “Please specify your ethnicity.” (Caucasian; Latino or Hispanic; Asian; Indigenous; Two or more; Other/unknown; Prefer not to answer)

4. “Please specify your educational and/or work-related background, prior to medicine.” (Natural sciences; Social sciences; Arts and culture; Science and technology; Languages; Mathematics; Business; Education; Caring profession)

5. “How many years have you been practicing family medicine?” (5 years or less; 6-10 years; 11-15 years; 16-20 years; 21 years or more)

6. “What is your current employment status?” (Full-time; Part-time; Seeking opportunities; Prefer not to answer)

7. “How often would you estimate that you typically use virtual care (e.g., telephone or video calls) in your day-to-day practice?” (Very infrequently; Somewhat infrequently; Occasionally; Somewhat frequently; Frequently; Very frequently)

**Scales**

**Comprehensive Relative Autonomy Index (C-RAI)**

The following questions relate to your reasons for using virtual healthcare in your practice. Different people have different reasons for doing this, and we want to know how true each of the following reasons are for you. Please use the following scale to indicate how true each reason is:

1 2 3 4 5 6 7

Not Somewhat Very

True True True

I am using virtual healthcare in my practice, because…

Amotivation:

1. AMO1: . . . I once had good reasons to use it, but now I don’t
2. AMO2: . . . Honestly, I don’t know why I am using it
3. AMO3: . . . I’m not sure, I wonder whether I should continue trying to use it
4. AMO4: . . . I used to know why I was using it, but I don’t anymore

External:

1. EXT1: . . . because important people will like me better if I do
2. EXT2: . . . because if I don’t use it, others will get mad
3. EXT3: . . . because I’ll get in trouble if I don’t use it
4. EXT4: . . . because I don’t have any choice but to use it

Negative introjection:

1. IJN1: . . . because I would feel guilty if I didn’t use it
2. IJN2: . . . because I would feel ashamed if I didn’t use it
3. IJN3: . . . because I would feel like a failure if I didn’t use it
4. IJN4: . . . because I don’t want to feel bad about myself

Positive introjection:

1. IJP1: . . . because I want to feel proud of myself
2. IJP2: . . . because I want to prove to myself that I am capable
3. IJP3: . . . because it boosts my self-esteem
4. IJP4: . . . because I want to feel good about myself

Identification:

1. IDE1: . . . because I strongly value using it
2. IDE2: . . . because using it is personally important to me
3. IDE3: . . . because it is my personal choice to use it
4. IDE4: . . . because using it is meaningful to me

Intrinsic:

1. INT1: . . . because I enjoy using it
2. INT2: . . . because using it is fun
3. INT3: . . . because it is a pleasure to use it
4. INT4: . . . because using it is interesting

**Basic Psychological Need Satisfaction and Frustration Scale-Work Domain (BPNSF-W)**

The following questions concern your feelings about your job during the PAST 4 WEEKS. Please indicate how much you agree with each of the following statements given your experiences on this job. Remember that your supervisor will never know how you responded to the questions.

1 2 3 4 5 6 7

Strongly Neutral Strongly

disagree agree

1. At work, I feel a sense of choice and freedom in the things I undertake.

2. I feel excluded from the group I want to belong to at work.

3. I feel confident that I can do things well on my job.

4. I feel that the people I care at work about also care about me.

5. Most of the things I do on my job feel like “I have to”.

6. When I am at work, I have serious doubts about whether I can do things well.

7. I feel that my decisions on my job reflect what I really want.

8. I feel that people who are important to me at work are cold and distant towards me.

9. At work, I feel capable at what I do.

10. I feel forced to do many things on my job I wouldn’t choose to do.

11. I feel disappointed with my performance in my job.

12. I feel connected with people who care for me at work, and for whom I care at work.

13. I feel my choices on my job express who I really am.

14. When I am at work, I feel competent to achieve my goals.

15. I feel pressured to do too many things on my job.

16. At work, I feel close and connected with other people who are important to me.

17. I feel insecure about my abilities on my job.

18. My daily activities at work feel like a chain of obligations.

19. I feel I have been doing what really interests me in my job.

20. I have the impression that people I spend time with at work dislike me.

21. In my job, I feel I can successfully complete difficult tasks.

22. I feel the relationships I have at work are just superficial.

23. When I am working, I feel like a failure because of the mistakes I make.

24. I experience a warm feeling with the people I spend time with at work.

Autonomy satisfaction (ASAT): items # 1, 7, 13, 19

Autonomy frustration (AFRU): items # 5, 10, 15, 18

Competence satisfaction (CSAT): items # 3, 9, 14, 21

Competence frustration (CFRU): items # 6, 11, 17, 23

Relatedness satisfaction (RSAT): items # 4, 12, 16, 24

Relatedness frustration (RFRU): items # 2, 8, 20, 22
